# Impact of IDH1 c.315C>T SNP on Outcomes in Acute Myeloid Leukemia: A Propensity Score-Adjusted Cohort Study

**DOI:** 10.3389/fonc.2022.804961

**Published:** 2022-03-18

**Authors:** Elizabeth M. Corley, Moaath K. Mustafa Ali, Hanan Alharthy, Kathryn A. F. Kline, Danielle Sewell, Jennie Y. Law, Seung Tae Lee, Sandrine Niyongere, Vu H. Duong, Maria R. Baer, Ashkan Emadi

**Affiliations:** ^1^ School of Medicine, University of Maryland, Baltimore, Baltimore, MD, United States; ^2^ Greenebaum Comprehensive Cancer Center, University of Maryland, Baltimore, Baltimore, MD, United States; ^3^ Department of Medicine, School of Medicine, University of Maryland, Baltimore, Baltimore, MD, United States; ^4^ Translational Genomics Laboratory, Greenebaum Comprehensive Cancer Center, University of Maryland, Baltimore, Baltimore, MD, United States; ^5^ Department of Pharmacology, School of Medicine, University of Maryland, Baltimore, Baltimore, MD, United States

**Keywords:** AML, IDH1 c.315C>T SNP, prognosis, survival, Myeloid mutations

## Abstract

Acute myeloid leukemia (AML) is the common type of acute leukemia in adults. Definitive prognostic significance of variants of unknown significance lacks for many commonly mutated genes, including the isocitrate dehydrogenase 1 (IDH1) synonymous single nucleotide polymorphism (SNP) variant c.315C>T. In this retrospective cohort study of 248 AML patients at the University of Maryland Greenebaum Comprehensive Cancer Center, we show that the IDH1 c.315C>T SNP, previously reported to be associated with poor prognosis by other studies with conflicting data, does not confer worse prognosis, with a median overall survival (OS) of 17.1 months compared to 15.1 months for patients without this SNP (P=0.57). The lack of negative effect on prognosis by IDH1 SNP c.315C>T is consistent with the absence of amino acid alteration (p.Gly105Gly).

## Introduction

It is estimated that 20,050 new cases of acute myeloid leukemia (AML) will be diagnosed in the United States in 2022 (1). AML-related mortality remains high, with an estimated 11,540 deaths expected in 2022 (1). Over the last decade, there have been significant advances in understanding the genetic landscape and pathophysiology of AML, leading to the approval of nine new medications for AML treatment (2). Targeted therapies are now available against the Fms-like tyrosine kinase 3 gene (FLT3) and isocitrate dehydrogenase (IDH) 1 and 2 gene mutations in AML (3).

The detection of genetic variants has become essential in determining risk stratification of AML and may guide treatment. However, definitive information on prognostic significance of various well-characterized mutations is still lacking. Although *FLT3-*ITD, Nucleophosmin-1 gene *(NPM1)* and CCAT/enhancer binding protein a gene (*CEBPA)* mutations have become established as prognostic markers in cytogenetically normal AML (CN-AML), there is a large group of patients without these mutations (4). Thus, there is a need for additional markers that may predict the differential outcomes of these patients. One potential source for expanding prognostication of AML is variants of unknown significance (VUS), such as common single nucleotide polymorphisms (SNPs) found in the population. A VUS becomes classified as pathogenic or benign once its impact is better understood. Therefore, studying VUS in commonly mutated genes may improve risk stratification and prognostication for AML patients.

Pathogenic variants within *IDH1* or *IDH2* occur in approximately 20% of AML (5). These mutations include R132 (in IDH1) and R140/R172 (in IDH2), which lead to the production of the oncometabolite 2-hydroxyglutarate and are targeted with selective oral inhibitors (6, 7). Currently, the VUS SNP in codon 105 in exon 4 of the *IDH1* gene (8), (c.315C>T (p.Gly105=), rs11554137), which occurs in approximately 5-10% of AML cases, is poorly understood. In two studies, the *IDH1* c.315C>T SNP was associated with an inferior outcome in cytogenetically normal AML (9, 10). In a third study, outcomes were also inferior, but this was attributable to association with *FLT3*-ITD (11). These studies proposed that the “silent” SNP may affect gene function by way of decreasing mRNA stability and thereby changing rates of protein translation, folding, and ultimately, function. However, these proposed ideas have never been demonstrated *in vitro* or in clinic. To date, the *IDH1* c.315C>T SNP is not commonly screened for in myeloid mutation panels. In this study, we hypothesized that presence of *IDH1* c.315C>T SNP does not impact clinical outcome of AML patients because of the lack of amino acid (glycine, Gly) change in position 105.

## Methods

### Study Design

We conducted a single-site retrospective cohort study to compare overall survival (OS), event-free survival (EFS) and complete remission (CR) and complete remission with incomplete hematologic recovery (CRi) rates in adults with AML with and without the *IDH1* c.315C>T (p.Gly105=) SNP from 2013 through 2020. OS was defined as the time from diagnosis to death from any cause. EFS was defined as the time from treatment initiation to induction failure, relapse, or death from any cause. Treatment response was evaluated according to the 2017 European LeukemiaNet (ELN) criteria (12). Composite CR rate included CR+CRi. The study was approved by the University of Maryland Baltimore Institutional Review Board (IRB).

### Data Source

We reviewed the medical records of patients diagnosed with AML at the University of Maryland Greenebaum Comprehensive Cancer Center (UMGCCC) between 2013 and 2020. UMGCCC uses the Epic electronic medical record (EMR) system. We used Epic and its features such as Care Everywhere and CRISP to extract relevant chart data from our site as well as all other available clinical sites within University of Maryland Medical System. The Care Everywhere feature allows access to a health network connecting all hospitals that utilize Epic. CRISP is a state-designed Health Information Exchange for a Maryland online database to extract relevant data from other clinical sites (13). We included all patients whose blood or bone marrow aspirate were examined for IDH mutation with Sanger sequencing which started at UMGCCC in 2013; no exclusion was performed. Data were collected and managed using Research Electronic Data Capture (REDCap) electronic data capture tools hosted at the University of Maryland (14, 15).

### Variables and Comparison Groups

Data extracted included age, gender, ethnicity, Eastern Cooperative Oncology Group (ECOG) performance status, baseline comorbidities, AML categories (*de novo* AML, myelodysplasia-related AML, myeloproliferative-related AML, therapy-related AML), cytogenetics, myeloid mutations including *IDH1*/*IDH2*/*FLT3/Tumor Protein 53 (TP53)*, treatments received, and outcomes. Data were checked multiple times by independent data collectors. We compared patients with and without the *IDH1* c.315C>T (p.Gly105=) SNP.

### Molecular Testing

#### Analysis of IDH1 and IDH2 Gene Alterations

*IDH1* and *IDH2* gene alterations were investigated using Sanger DNA sequencing. *IDH1* Codon 132, *IDH2* codons 140 and 172, and the surrounding sequences within exon 4 were analyzed on whole blood or bone marrow aspirate by Sanger DNA sequencing on an Applied Biosystems 3730XL genetic analyzer, using Sequencher™ DNA Sequence Analysis Software (version 5). The c.315C>T SNP in *IDH1* codon 105 is in the same exon as the R132 mutation. These sequences were compared to NCBI reference sequences for the *IDH1* (NM_005896.3 and NP_005887.2) and *IDH2* (NM_002168.3 and NP_002159.2) genes. The lower limit of detection for this assay is approximately 20% allele proportion.

### Propensity Score Estimation

This study obtained the Average Treatment Effect on the Treated (ATT) (16). We included the following variables in the propensity score model: age at diagnosis, gender, ethnicity, comorbidities, ECOG performance status, type of AML, cytogenetics at diagnosis, *FLT3*, *IDH1*, *IDH2* and *TP53* mutational status and first-line treatment. Different methods for matching were attempted, including 1:1 nearest neighbor, 1:2 nearest neighbor, full matching, inverse probability weighting, and weighting by the odds. Full matching was chosen as the matching method because it achieved the lowest standardized biases differences, smallest coefficients of variations and smallest weights. Weights obtained from full matching were used to adjust outcomes. No patients were dropped in the matching process. The choice of estimand (ATT) was based on achieving standardized bias scores less than 0.25 (17). We used balance tables and Love plots to assess for covariate balance before and after matching. As a sensitivity check, we repeated the analysis using inverse probability weighting and obtained average treatment effect as an estimand. Generalized boosted model was used to calculate weights. The results of inverse probability weighting are provided as supplementary data. Cluster-robust standard errors were used to account for subclass membership in the matching process. The R statistical package “MatchIt” and “WeightIt” were used for propensity score weighting (18).

### Statistical Analysis

Descriptive statistics were used to compare baseline characteristics of patients with and without *IDH1* c.315C>T. Categorical variables were presented as absolute numbers and percentages. Continuous variables were presented as means with standard deviations or medians with interquartile ranges (IQR). Baseline characteristics were compared using Pearson chi-square or Fisher’s exact test when categorical or t-test when continuous. OS and EFS were compared using log-rank and Gehan Breslow-Wilcoxon rank tests. Multivariable and univariable Cox proportional hazards models were used to assess relative mortality. Regression diagnostics were used to evaluate model assumptions. All statistical tests were two-sided, and P-values <0.05 were considered statistically significant. R-statistical software (version 4.1.1) was used for statistical analyses.

## Results

### Cohort Characteristics

We identified a total of 444 AML patients treated at UMGCCC during the study period that we had Sanger sequencing data on patients (2013–2020). All patients tested for IDH1 mutations using the Sanger technique were included (2015-2020). Patients not tested for *IDH1* mutations were excluded; ultimately, 248 patients were included. There was no other exclusion criteria. The median age was 65 years [IQR 54-75] and 42% were female. Median follow-up was 27.33 months [IQR 17.6-46.9]. Median OS for the whole population was 17.1 months (CI 13.8-21.8). The *IDH1* c.315C>T SNP was found in 23 patients (9%). **Table 1** shows propensity score-adjusted baseline characteristics in patients with and without the *IDH1* c.315C>T SNP. After matching, there were no statistically significant differences in baseline characteristics between the two groups. Covariate balance before and after propensity score weighting is shown in **Supplemental Figure 1**. Unadjusted baseline characteristics are shown in **Supplementary Table 1**. In the unadjusted cohort, patients with the *IDH1* c.315C>T SNP compared to patients without received the following treatments: intensive chemotherapy (30% vs. 34%), hypomethylating agent with or without others treatments (30% vs. 21%), hypomethylating agents with venetoclax (22% vs. 18%), clinical trial (9% vs. 19%), other treatments (0 vs. 4%) and none (9% vs. 4%).

**Table 1:** Adjusted baseline characteristics of patients with IDH1 c.315C>T mutated vs. IDH1 wild-type AML.

	IDH1 c.315C>T Mutated	Percentage/SD/IQR	IDH1 wild-type AML	Percentage/SD/IQR	P-value
**Number**	23	–	51^β^		
**Female**	8	0.35	19	0.37	0.85
**Ethnicity**			0	0	0.85
**Caucasian**	13	0.57	30	0.59	
**Other**	10	0.43	21	0.41	
**Unknown**	0	0	0	0	
**Comorbidities**					
**Cardiovascular disease**	6	0.26	11	0.21	0.65
**Diabetes mellitus**	7	0.3	18	0.36	0.53
**Hypertension**	11	0.48	21	0.41	0.66
**CKD stage III-V/ESRD**	1	0.04	2	0.04	0.95
**Asthma/COPD**	4	0.17	8	0.15	0.77
**Active Cancer**	2	0.09	3	0.06	0.54
**AML type**					0.96
**AML, *de novo***	15	0.65	33	0.65	
**AML with MDS/CMML changes**	4	0.17	9	0.17	
**AML with prior MPN**	2	0.09	6	0.11	
**Therapy-Related AML**	2	0.09	4	0.07	
**Cytogenetic Category**					0.98
**Favorable Risk**	1	0.04	2	0.04	
**Intermediate Risk**	29	0.82	43	0.83	
**Unfavorable Risk**	2	0.09	4	0.07	
**Not performed or Inadequate**	1	0.04	3	0.06	
**IDH1 mutated**	2	0.09	7	0.14	0.65
**IDH2 mutated**	3	0.13	6	0.12	0.93
**FLT3-ITD status**					0.99
**FLT3-ITD mutated 1-49%**	1	0.04	2	0.04	
**FLT3-ITD mutated 50-100%**	1	0.04	2	0.039	
**FLT3 WT**	19	0.83	42	0.83	
**Not tested**	2	0.09	5	0.09	
**FLT3-TKD status**					0.95
**FLT3-TKD mutated**	4	0.17	8	0.15	
**FLT3 WT**	17	0.74	39	0.76	
**Not tested**	2	0.09	5	0.09	
**P53 status**					0.98
**P53 mutated**	3	0.13	7	0.14	
**P53 WT**	8	0.35	17	0.33	
**Not tested**	12	0.52	27	0.53	
**ECOG status III/IV**	1	0.04	4	0.08	0.6
**First treatment received**					0.95
**Anthracycline-based regimen**	8	0.35	16	0.32	
Other*	13	0.57	31	0.6	
**None**	2	0.09	4	0.08	
**Age (Average ± SD)**	65.6	15.9	66.5	15.2	0.81
**Age (Median, IQR)**	68.5	59.6-77.7	69.1	58.1-77.9	0.8

^β^Estimated sample size, the unweighted control number is 225 patients. *Other therapies include but are not limited to Venetoclax, decitabine, and cytarabine regimens. N.B. In this propensity-score model, no patients were excluded from the analysis. FLT3, fms-like tyrosine kinase 3; ITD, internal tandem duplication; TKD, tyrosine kinase domain; WT, wild type.

### Outcomes

**Figure 1:**
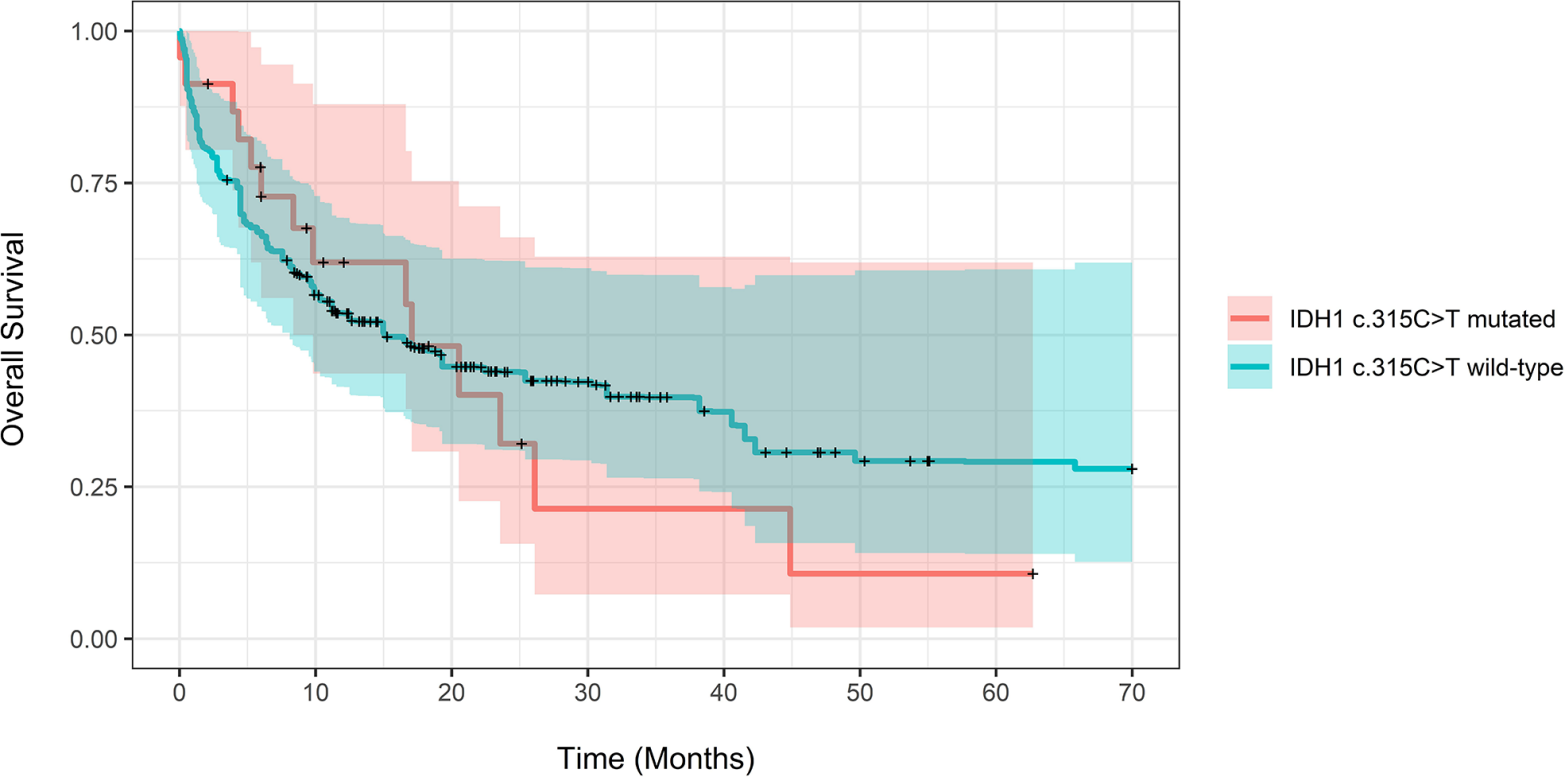
Propensity score-adjusted Overall Survival for patients with IDH1 c.315C>T mutated vs. IDH1 wild-type AML. Log-Rank adjusted P-Value was non-significant (P = 0.57).

Excluding patients who did not have a bone marrow biopsy, the adjusted composite CR rate for patients with and without the *IDH1* c.315C>T SNP was 77.10% compared to 65.30%; this finding was not statistically significant (P=0.53). The death at the end of observation in the IDH mutated group was 14 patients (39.10%) vs. 81 in the wildtype group (36%); P=0.944. The adjusted median OS for patients with compared to without the *IDH1* c.315C>T SNP was 17.1 months (CI 8.37-Not calculable (NC)) compared to 15.1 months (CI 8.1-77.3, P=0.57). The unadjusted median OS for patients with and without the *IDH1* c.315C>T SNP was 17.1 months (CI 9.8-NC) and 17 months (CI 13-22.2) (P=0.9). Adjusted OS difference at years 1-3 for patients with and without the *IDH1* c.315C>T SNP showed no statistically significant difference (**Supplementary Table 2** **)**. On weighted-univariable Cox proportional hazards regression of the total cohort, there was no statistically significant difference in relative mortality (HR 1.08, CI 0.62-1.93, P=0.79). **Figure 1** demonstrates propensity score-adjusted OS for patients with and without the *IDH1* c.315C>T SNP.

**Figure 2:**
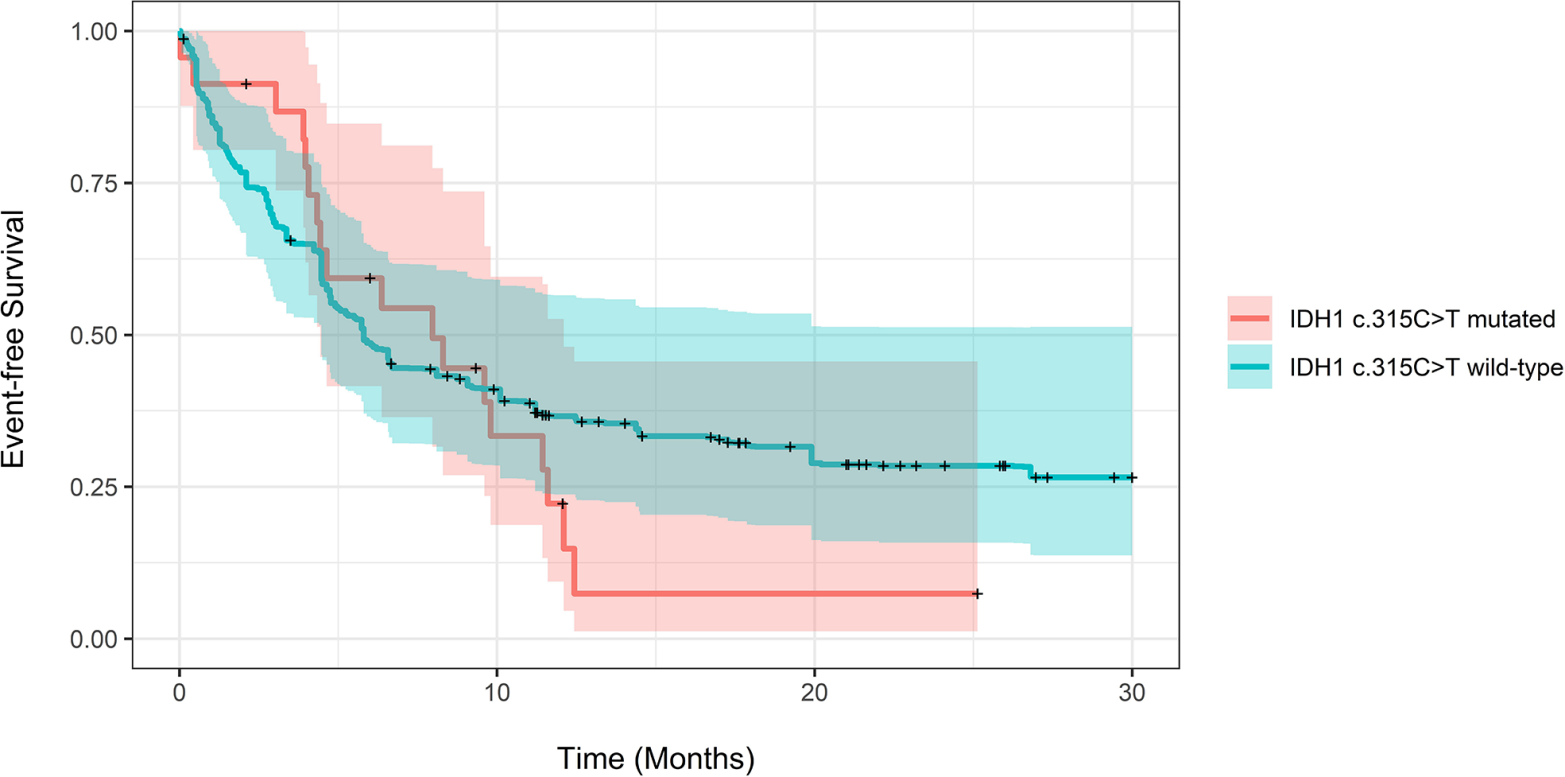
Propensity score-adjusted Event-free Survival for patients with IDH1 c.315C>T mutated vs. IDH1 wild-type AML. Log-Rank adjusted P-Value was non-significant (P = 0.73).

The adjusted median EFS for patients with and without the *IDH1* c.315C>T SNP was 5.8 months (CI 4.47-74) compared to 7.97 (CI: 4.43-12.1, P=0.73). Adjusted EFS at years 1-3 for patients with and without the SNP also showed no statistical significance (**Supplementary Table 3**). The relative mortality and progression were not different in patients with and without the SNP (HR 1.18, CI 0.71-1.98, P=0.5) using Cox proportional hazards regression. **Figure 2** demonstrates propensity score-adjusted EFS for patients with vs. without the IDH1 c.315C>TSNP. As a sensitivity check, we repeated analysis using inverse probability weighting. There was no statistically significant difference in adjusted median OS or median EFS between the two groups. The results are provided in supplementary file (**Supplementary Tables 4–6** and **Supplementary Figures 2–4**).

## Discussion

Prognostic models for AML largely rely on cytogenetic aberrations and somatic mutations such as *FLT3*-ITD and *NPM1* and *CEBPA* mutations in patients with a normal karyotype (4). However, many intermediate-risk patients have poorly understood AML genetic profiles, which may hinder accurate prognostication and clinical decision-making. We, therefore, aimed to study the *IDH1* c.315C>T SNP, a poorly understood VUS seen in ~5-10% of AML cases (8, 9).

Previous data on the prognostic significance of this variant have been mixed; while it was shown to have inferior outcomes for cytogenetically normal AML in two studies (9, 10), another study showed inferior outcomes that were attributable to association with *FLT3*-ITD (11). Of the two studies that showed the adverse prognostic significance of the *IDH1* c.315C>T SNP, one (10) (N=51, 8 with variant) reported that the SNP confers an inferior prognosis in *NPM1*/*CEBPA* wild-type Egyptian patients with AML. The other study (9) showed that the SNP had a negative effect on outcomes in univariate, but not multivariate, analysis, with the greatest impact in *NPM1*/*FLT3* high-risk patients (either *NPM1* wild-type or *FLT3*-ITD). These studies proposed that the synonymous SNP may induce genetic alteration at the mRNA level, such as alterations mRNA stability, folding, or splicing; however, all these studies were vulnerable to inadequate design (10). In order to evaluate such potential mechanisms, future RNA-seq studies to analyze transcriptome profile and to characterize changes in ribosome-associated mRNA (i.e. translatome) are warranted.

We hypothesized that due to the synonymous nature of *IDH1* c.315C>T mutation resulting in uninterrupted presence of glycine in position 105 of the protein, the biochemical function of IDH1 enzyme remains intact; hence has no impact on the clinical outcome of patient. In this report, we confirm our hypothesis. Compared to prior studies, our study had greater power, with a large sample size (N=248), more patients with the variant (23 patients, 9%), and adjustment for more extensive disease profile data, allowing for many variables to be controlled. In addition, our study used propensity score weighting to adjust for baseline confounding, which showed no statistical prognostic difference between cohorts while controlling for baseline characteristics, including *FLT3* mutations. This is consistent with the previous multivariate analysis (9). Our study adds to the current data, revealing that with greater power and tight statistical control of an extensive number of confounding variables, there was no negative prognostic value for the *IDH1* c.315C>T SNP, with statistically insignificant differences in clinical outcome.

The major limitation of this study is that it was a retrospective single-site model. To control for observable confounding variables, we adjusted outcomes using propensity score analysis. After matching, the standardized mean difference was less than 0.2 in all variables. As a sensitivity analysis, we conducted weighted-multivariable Cox proportional hazards regression to adjust for possible remaining confounding, and there was no qualitative difference in outcomes.

## Conclusion

Our retrospective cohort study showed that, unlike in previous studies and concordant with our mechanism-based hypothesis, the presence of *IDH1* c.315C>T SNP was not associated with inferior OS, PFS or CR+CRi rates compared with its absence. Due to the rarity of this SNP, further collaborative study with multiple institutions is warranted to understand the impact of this SNP fully.

## Data Availability Statement

The data that support the findings of this study are available from MMA, Moaath.mustafaali@umm.edu, upon reasonable request.

## Ethics Statement

The studies involving human participants were reviewed and approved by University of Maryland Institutional Review Board. Written informed consent for participation was not required for this study in accordance with the national legislation and the institutional requirements.

## Author Contributions

The authors confirm their contribution to the paper as follows: Study conception and design: EC, MMA, and AE. Data Collection: EC, MMA, HA, KK, and DS. Analysis and Interpretation: EC, MMA, HA, KK, DS, JL, SL, SN, VD, MB, and AE. Draft manuscript preparation: EC, MMA, and AE. Statistical analysis: MMA. Critical Review of Manuscript: HA, KK, DS, JL, SL, SN, VD, MB, and AE. Administrative and technical support: MMA. Supervision: MMA and AE. All authors reviewed the results and approved the final version of the manuscript.

## Funding

This research was supported by funds through the National Cancer Institute - Cancer Center Support Grant (CCSG) - P30CA134274 and through the Maryland Department of Health’s Cigarette Restitution Fund Program.

## Conflict of Interest

The authors declare that the research was conducted in the absence of any commercial or financial relationships that could be construed as a potential conflict of interest.

## Publisher’s Note

All claims expressed in this article are solely those of the authors and do not necessarily represent those of their affiliated organizations, or those of the publisher, the editors and the reviewers. Any product that may be evaluated in this article, or claim that may be made by its manufacturer, is not guaranteed or endorsed by the publisher.
