# Supplementary material for: Impact of IDH1 c.315C>T SNP on Outcomes in Acute Myeloid Leukemia: A Propensity Score-Adjusted Cohort Study
Source: Front Oncol. 2022 Mar 18;12:804961. doi: 10.3389/fonc.2022.804961 (PMC8972959; doi:10.3389/fonc.2022.804961)
Supplement: Supplementary file 1 [file DataSheet_1.docx]

Supplementary Material

# Supplementary Figures and Tables

## Supplementary Table 1. Unadjusted baseline characteristics of patients with *IDH1* c.315C>T mutated vs. *IDH1* wild-type AML

|  | ***IDH1* c.315C>T Mutated** | **Percentage/ SD/ IQR** | ***IDH1* Wild-Type** | **Percentage/ SD/ IQR** | **P-value** |
| --- | --- | --- | --- | --- | --- |
| **Number** | 23 | - | 225 |  |  |
| **Female** | 8 | 0.35 | 96 | 0.43 | 0.61 |
| **Ethnicity** |  |  |  |  | 0.52 |
| **Caucasian** | 13 | 0.57 | 152 | 0.68 |  |
| **Other** | 10 | 0.43 | 72 | 0.32 |  |
| **Unknown** | 0 | 0.00 | 1 | 0.00 |  |
|  |  |  |  |  |  |
| **Comorbidities** |  |  |  |  |  |
| **Cardiovascular disease** | 6 | 0.26 | 52 | 0.23 | 0.95 |
| **Diabetes mellitus** | 7 | 0.30 | 48 | 0.21 | 0.46 |
| **Hypertension** | 11 | 0.48 | 99 | 0.44 | 0.90 |
| **CKD stage III-V/ESRD** | 1 | 0.04 | 10 | 0.04 | 1.00 ^μ^ |
| **Asthma/ COPD** | 4 | 0.17 | 21 | 0.09 | 0.39 |
| **Active Cancer** | 2 | 0.09 | 7 | 0.03 | 0.44 |
|  |  |  |  |  |  |
| **AML type** |  |  |  |  | 0.59 |
| **AML, de novo** | 15 | 0.65 | 125 | 0.56 |  |
| **AML with MDS/ CMML changes** | 4 | 0.17 | 61 | 0.27 |  |
| **AML with prior MPN** | 2 | 0.09 | 11 | 0.05 |  |
| **Therapy-Related AML** | 2 | 0.09 | 28 | 0.12 |  |
|  |  |  |  |  |  |
| **Cytogenetic Category** |  |  |  |  | 0.96 |
| **Favorable Risk** | 1 | 0.04 | 15 | 0.07 |  |
| **Intermediate Risk** | 19 | 0.82 | 174 | 0.78 |  |
| **Unfavorable Risk** | 2 | 0.09 | 27 | 0.12 |  |
| **Not performed or Inadequate** | 3 | 0.13 | 8 | 0.03 |  |
|  |  |  |  |  |  |
| ***IDH1* mutated** | 2 | 0.09 | 16 | 0.07 | 1.00^μ^ |
| ***IDH2* mutated** | 3 | 0.13 | 34 | 0.15 | 1.00 ^μ^ |
|  |  |  |  |  |  |
| ***FLT3*-ITD status** |  |  |  |  | 0.84 |
| ***FLT3*-ITD mutated 1-49%** | 1 | 0.04 | 24 | 0.11 |  |
| ***FLT3*-ITD mutated 50-100%** | 1 | 0.04 | 17 | 0.08 |  |
| ***FLT3* WT** | 19 | 0.83 | 166 | 0.74 |  |
| **Not tested** | 2 | 0.09 | 18 | 0.08 |  |
|  |  |  |  |  |  |
| ***FLT3*-TKD status** |  |  |  |  | 0.99 |
| ***FLT3*-TKD mutated** | 4 | 0.17 | 41 | 0.18 |  |
| ***FLT3* WT** | 17 | 0.74 | 166 | 0.74 |  |
| **Not tested** | 2 | 0.09 | 18 | 0.08 |  |
|  |  |  |  |  |  |
| ***TP53* status** |  |  |  |  | 0.02 |
| ***TP53* mutated** | 3 | 0.13 | 36 | 0.16 |  |
| ***TP53* WT** | 8 | 0.35 | 134 | 0.60 |  |
| **Not tested** | 12 | 0.52 | 55 | 0.24 |  |
|  |  |  |  |  |  |
| **ECOG status III/IV** | 1 | 0.04 | 16 | 0.07 | 0.95 |
|  |  |  |  |  |  |
| **First treatment received** |  |  |  |  | 0.51 |
| **Anthracycline-based regimen** | 8 | 0.35 | 95 | 0.42 |  |
| **Other^*^** | 13 | 0.57 | 121 | 0.54 |  |
| **None** | 2 | 0.09 | 9 | 0.04 |  |
|  |  |  |  |  |  |
| **Age (Average ± SD)** | 65.6 | 15.9 | 62.3 | 15.8 | 0.36 |
| **Age (Median, IQR)** | 68.5 | 59.6-77.7 | 64.9 | 54-74.8 | 0.13 |

^μ^ Approximated to 1

*Other therapies include but are not limited to Venetoclax, decitabine, and cytarabine regimens. SD = standard deviation

**Supplementary Table 2**. Adjusted OS at years 1-3 for patients with *IDH1* c.315C>T mutated vs. *IDH1* wild-type AML

| **strata** | **Time (months)** | **Estimated survival (%)** | **Lower CI** | **Upper CI** |
| --- | --- | --- | --- | --- |
| *IDH1* c.315C>T mutated | 12 | 0.62 | 0.44 | 0.88 |
| *IDH1* c.315C>T mutated | 24 | 0.32 | 0.16 | 0.66 |
| *IDH1* c.315C>T mutated | 36 | 0.21 | 0.07 | 0.63 |
| *IDH1* c.315C>T wild-type | 12 | 0.54 | 0.41 | 0.69 |
| *IDH1* c.315C>T wild-type | 24 | 0.44 | 0.31 | 0.62 |
| *IDH1* c.315C>T wild-type | 36 | 0.40 | 0.26 | 0.60 |

**Supplementary Table 3.**  Adjusted EFS at years 1-3 for patients with and without *IDH1* c.315C>T mutation

| strata | Time (months) | Estimated survival (%) | Lower CI | Upper CI |
| --- | --- | --- | --- | --- |
| *IDH1* c.315C>T mutated | 12 | 0.22 | 0.09 | 0.53 |
| *IDH1* c.315C>T mutated | 24 | 0.07 | 0.01 | 0.46 |
| *IDH1* wild-type | 12 | 0.37 | 0.24 | 0.57 |
| *IDH1* wild-type | 24 | 0.28 | 0.16 | 0.51 |

**Supplementary Table 4:** Sensitivity Analysis, adjusted median OS in months for patients with *IDH1* c.315C>T mutated vs *IDH1*wild-type AML

|  | strata | Median  (months) | Lower CI | Upper CI | P-Value |
| --- | --- | --- | --- | --- | --- |
| Adjusted | | | | | |
| 1 | *IDH1* c.315C>T mutated | 20.5 | 9.8 | NA | 0.88 |
| 2 | *IDH1* wild-type | 17 | 13 | 22.3 |  |
| Unadjusted | | | | | |
| 1 | *IDH1* c.315C>T mutated | 17.1 | 9.8 | NA | 0.9 |
| 2 | *IDH1* wild-type | 17 | 13 | 22.3 |  |

**Supplementary Table 5:** Sensitivity analysis, Adjusted OS at years 1-3 for patients with *IDH1* c.315C>T mutated vs. *IDH1* wild-type AML

| strata | Time (months) | Estimated survival (%) | Lower CI | Upper CI |
| --- | --- | --- | --- | --- |
| *IDH1* c.315C>T mutated | 12 | 0.61 | 0.40 | 0.93 |
| *IDH1* c.315C>T mutated | 24 | 0.40 | 0.20 | 0.80 |
| *IDH1* c.315C>T mutated | 36 | 0.29 | 0.11 | 0.79 |
| *IDH1* wild-type | 12 | 0.59 | 0.52 | 0.65 |
| *IDH1* wild-type | 24 | 0.41 | 0.34 | 0.48 |
| *IDH1* wild-type | 36 | 0.33 | 0.26 | 0.42 |

**Supplementary Table 6:** Sensitivity Analysis, Adjusted median EFS in months for patients with *IDH1* c.315C>T mutated vs *IDH1* wild-type AML

|  | strata | Median  (months) | Lower CI | Upper CI | P-Value |
| --- | --- | --- | --- | --- | --- |
| 1 | *IDH1* c.315C>T mutated | 8.3 | 4.43 | NA | 0.69 |
| 2 | *IDH1* wild-type | 6 | 5.07 | 9.2 |  |

**Supplementary Figure 1: Covariate balance before and after propensity score full matching**


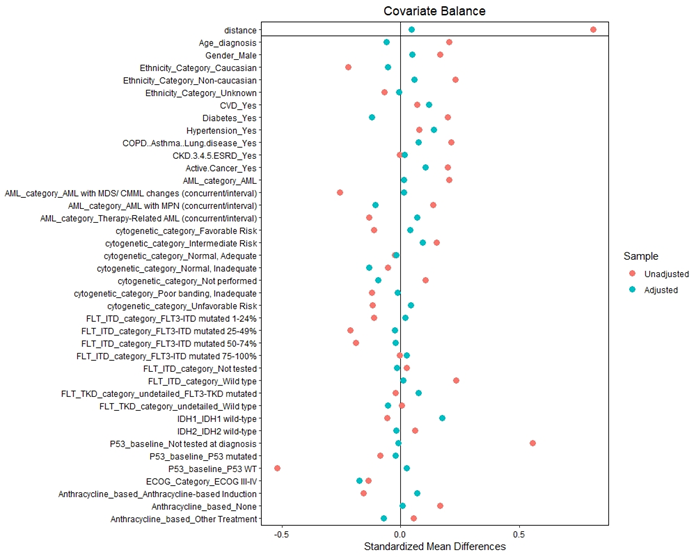


**Supplementary Figure 2:** Sensitivity analysis, covariate balance before and after propensity score weighting


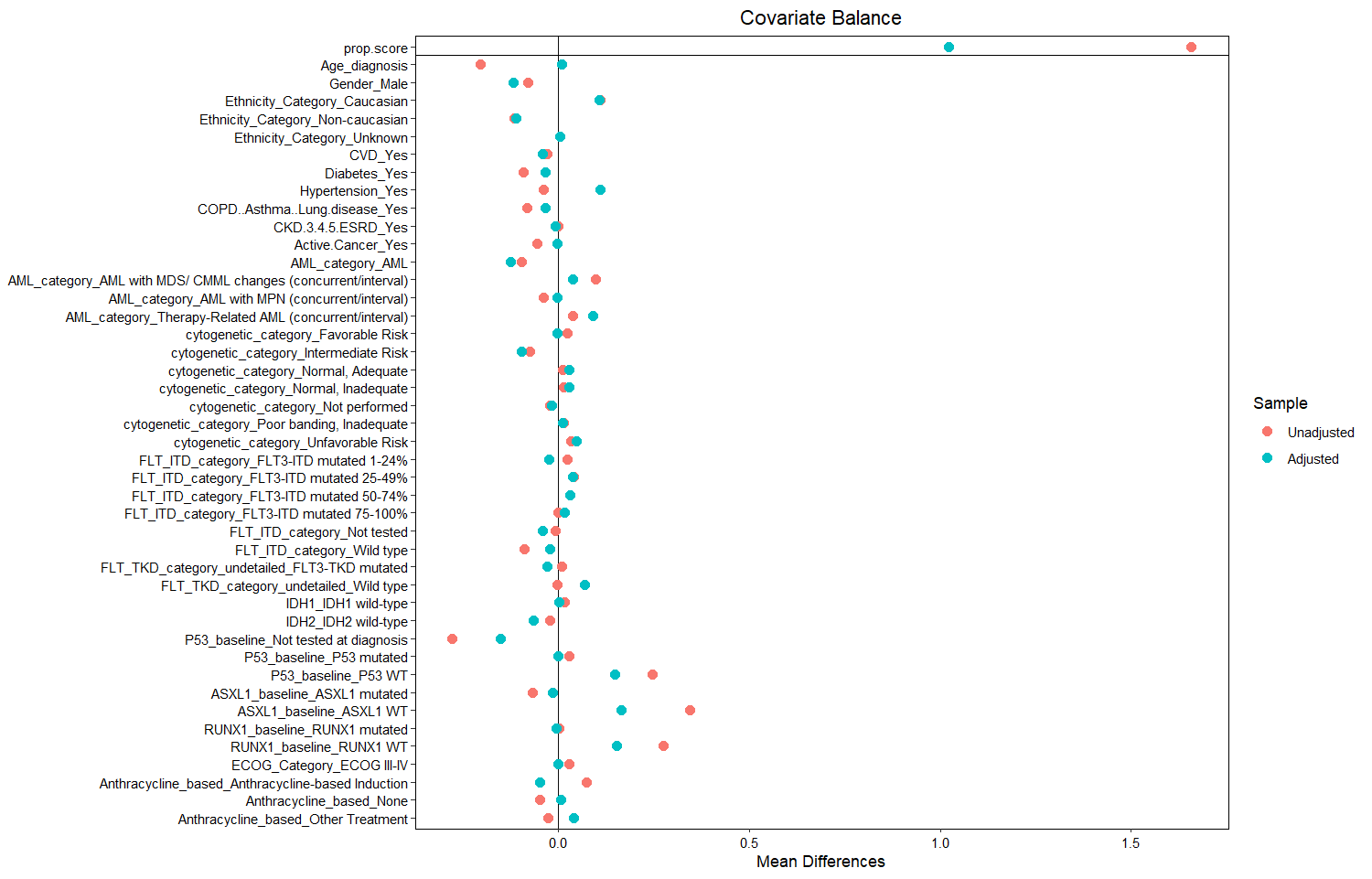


**Supplemental Figure 3**: Sensitivity analysis, propensity score-adjusted Overall Survival for patients with IDH1 c.315C>T mutated vs. IDH1 wild-type AML


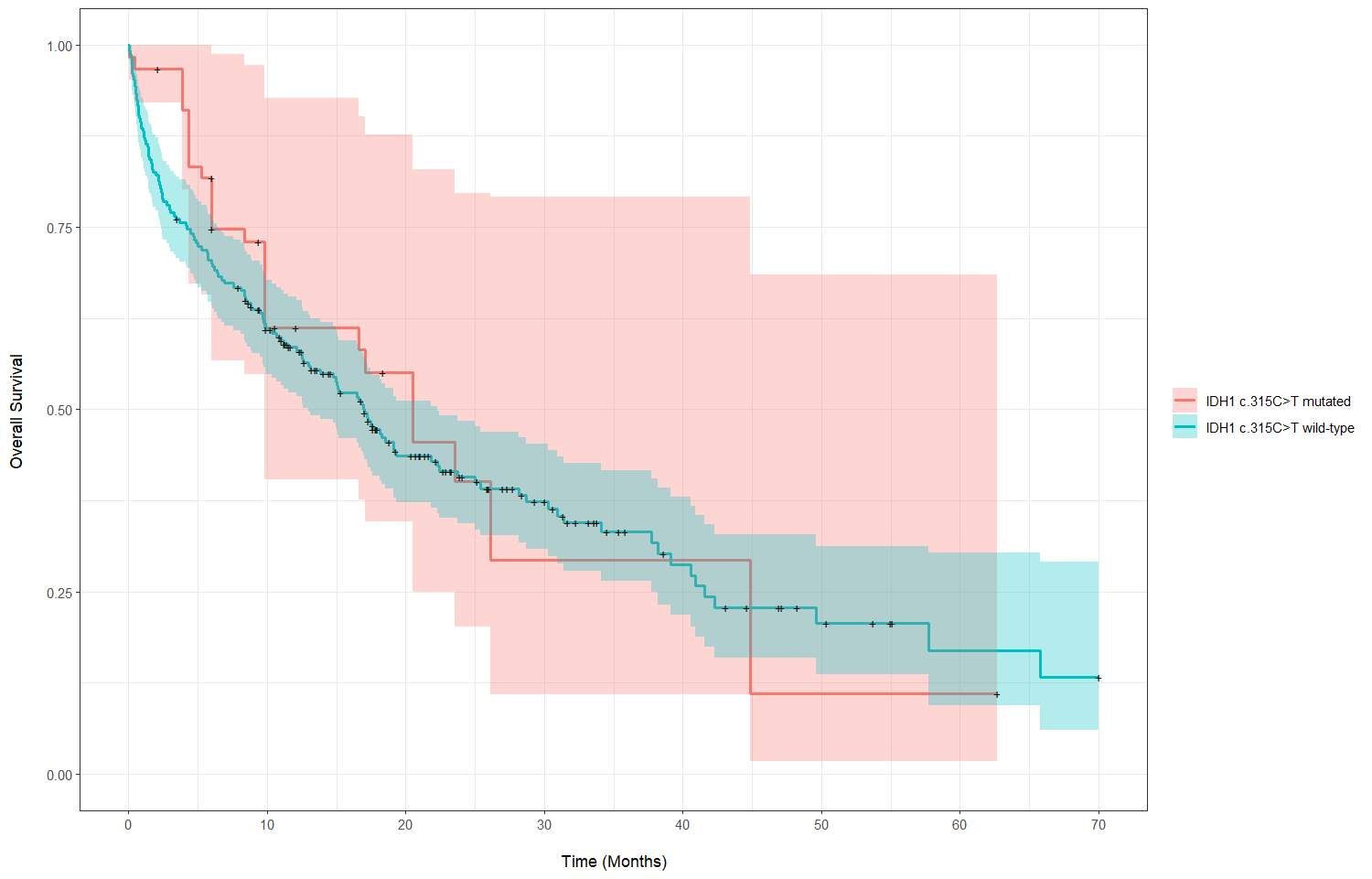


Log-Rank adjusted P-Value was non-significant.

**Supplemental Figure 4**: Sensitivity analysis, score-adjusted Event-free Survival for patients with IDH1 c.315C>T mutated vs. IDH1 wild-type AML


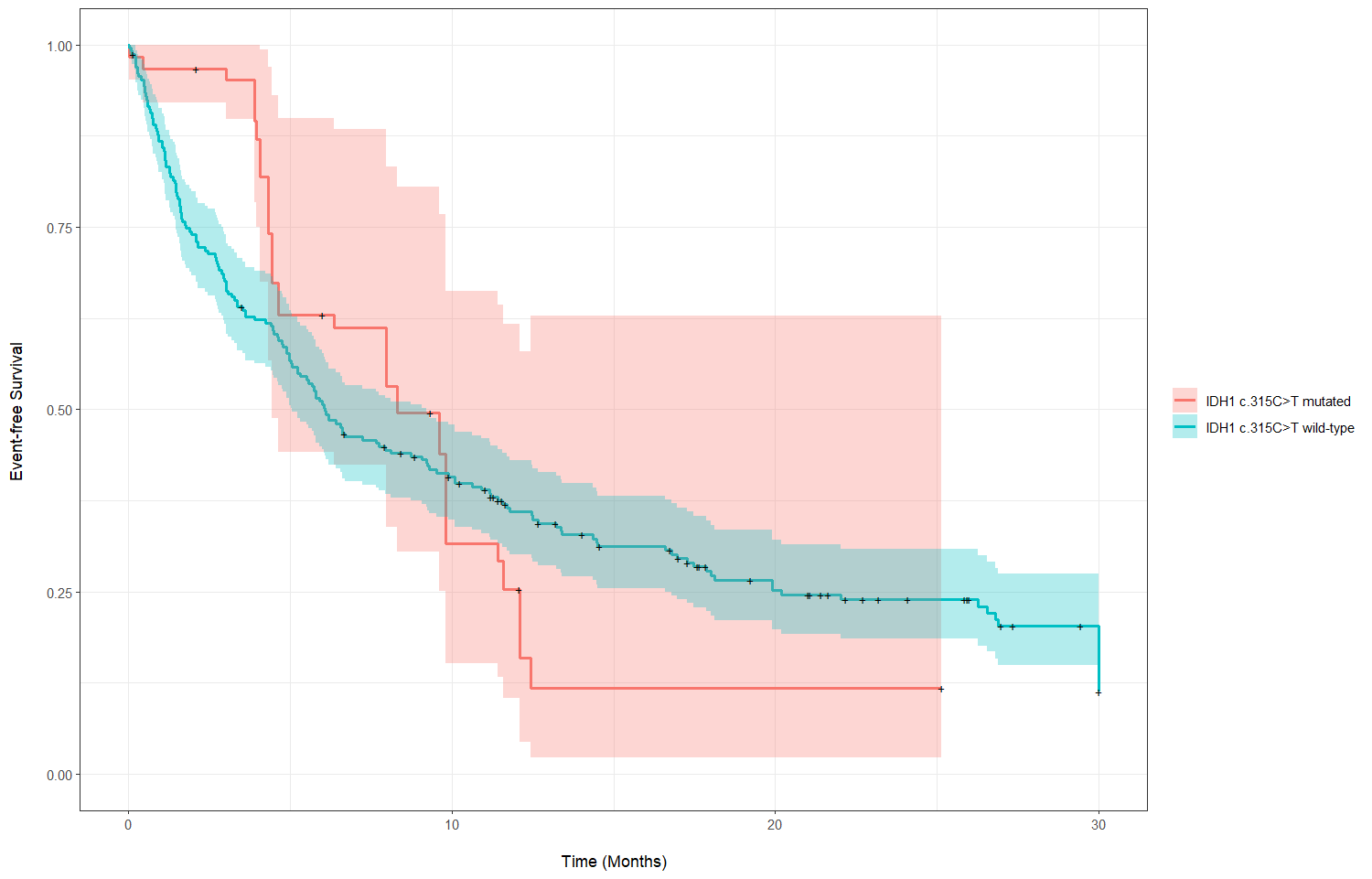


Log-Rank adjusted P-Value was non-significant.
